# Supplementary material for: Cryo‐EM structures of perforin‐2 in isolation and assembled on a membrane suggest a mechanism for pore formation
Source: EMBO J. 2022 Oct 17;41(23):e111857. doi: 10.15252/embj.2022111857 (PMC9713709; doi:10.15252/embj.2022111857)
Supplement: Supplementary file 1 — Appendix [file EMBJ-41-e111857-s009.pdf]

APPENDIX

Cryo-EM structures of perforin-2 in isolation and assembled on a membrane suggest a mechanism for pore formation

Xiulian Yu[1,2], Tao Ni[1,5], George Munson[5], Peijun Zhang[1,3,4,5], Robert J. C. Gilbert[1,2,5]

[1]Division of Structural Biology, Wellcome Centre for Human Genetics, University of Oxford, Roosevelt Drive, Oxford OX3 7BN

[2]Calleva Research Centre for Evolution and Human Sciences, Magdalen College, University of Oxford, Oxford, OX1 4AU, UK

[3]Diamond Light Source, Harwell Science and Innovation Campus, Didcot OX11 0DE, UK

[4]Chinese Academy of Medical Sciences Oxford Institute, University of Oxford, Oxford, OX3 7BN, UK

[5]To whom correspondence should be addressed: tao.ni@strubi.ox.ac.uk, peijun.zhang@strubi.ox.ac.uk, robert.gilbert@magd.ox.ac.uk

Appendix Figure S1: Structure of mPFN2 on membrane and comparison with hPFN2 structures.

Appendix Figure S2: Comparison of PFN2 structures with other MACPF/CDC pore-forming proteins in their pore conformation.

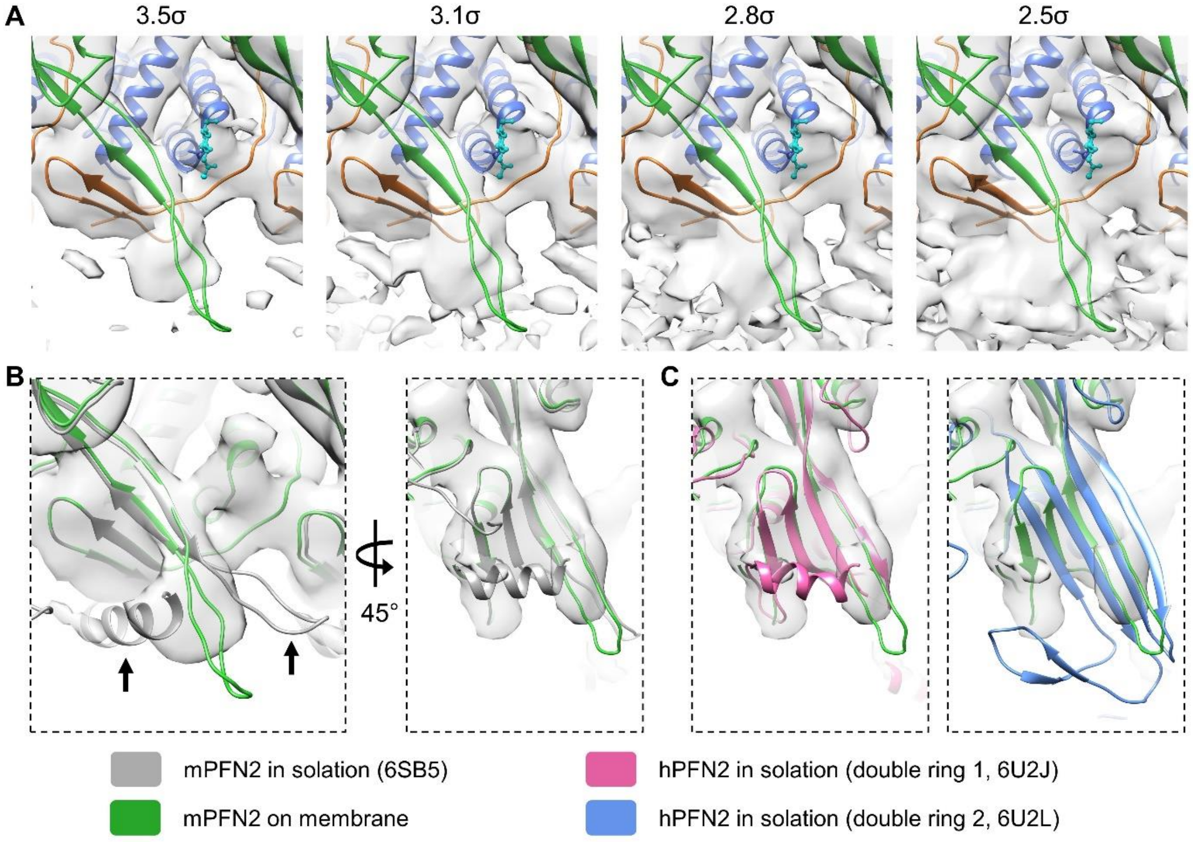

Appendix Figure S1: Structure of mPFN2 on a membrane and comparison with hPFN2 structures. (A) Structure of mPFN2 fit into cryoSTA density map at different thresholds, focusing on the region of the CTT and β-hairpin. The map sigma level was calculated based on density distribution of the whole volume in Chimera, without a molecular mask. (B) Overlay of mPFN2 structures in isolation and on a membrane with the cryoSTA density map from mPFN2 on the membrane. Black arrows point to the difference in two structures. (C) Overlay of hPFN2 structures in isolation (single ring and double ring conformations, PDB 6U2J and 6U2L) into the mPFN2 cryoSTA density map. hPFN2 in isolation from one of the double ring conformations can mostly fit into cryoSTA density map of mPFN2 on a membrane (pink), while the other double ring conformation does not (blue).

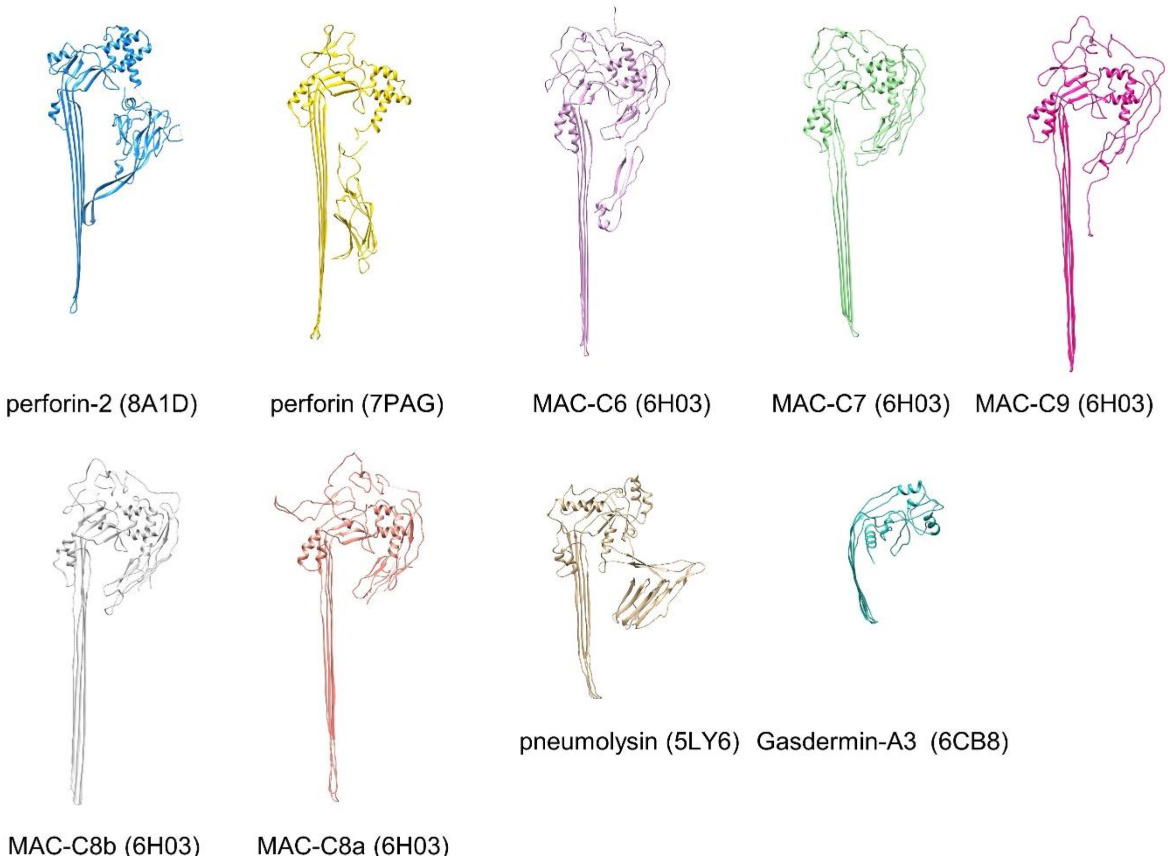

Appendix Figure S2: Comparison of PFN2 structures with other MACPF/CDC pore-forming proteins in their pore conformation. One subunit from each pore complex (PDB codes in brackets) was extracted and aligned to the perforin-2 structure. Perforin and MAC complex components (C6, C8a, C8b and C9) have longer transmembrane β-hairpins than perforin-2; while bacterial cholesterol-dependent cytolysins (pneumolysin) and gasdermin family pores have much shorter transmembrane β-hairpins.
