# Supplementary material for: Cryo‐EM structures of perforin‐2 in isolation and assembled on a membrane suggest a mechanism for pore formation
Source: EMBO J. 2022 Oct 17;41(23):e111857. doi: 10.15252/embj.2022111857 (PMC9713709; doi:10.15252/embj.2022111857)
Supplement: Supplementary file 2 — Expanded View Figures PDF [file EMBJ-41-e111857-s007.pdf]

Expanded View Figures

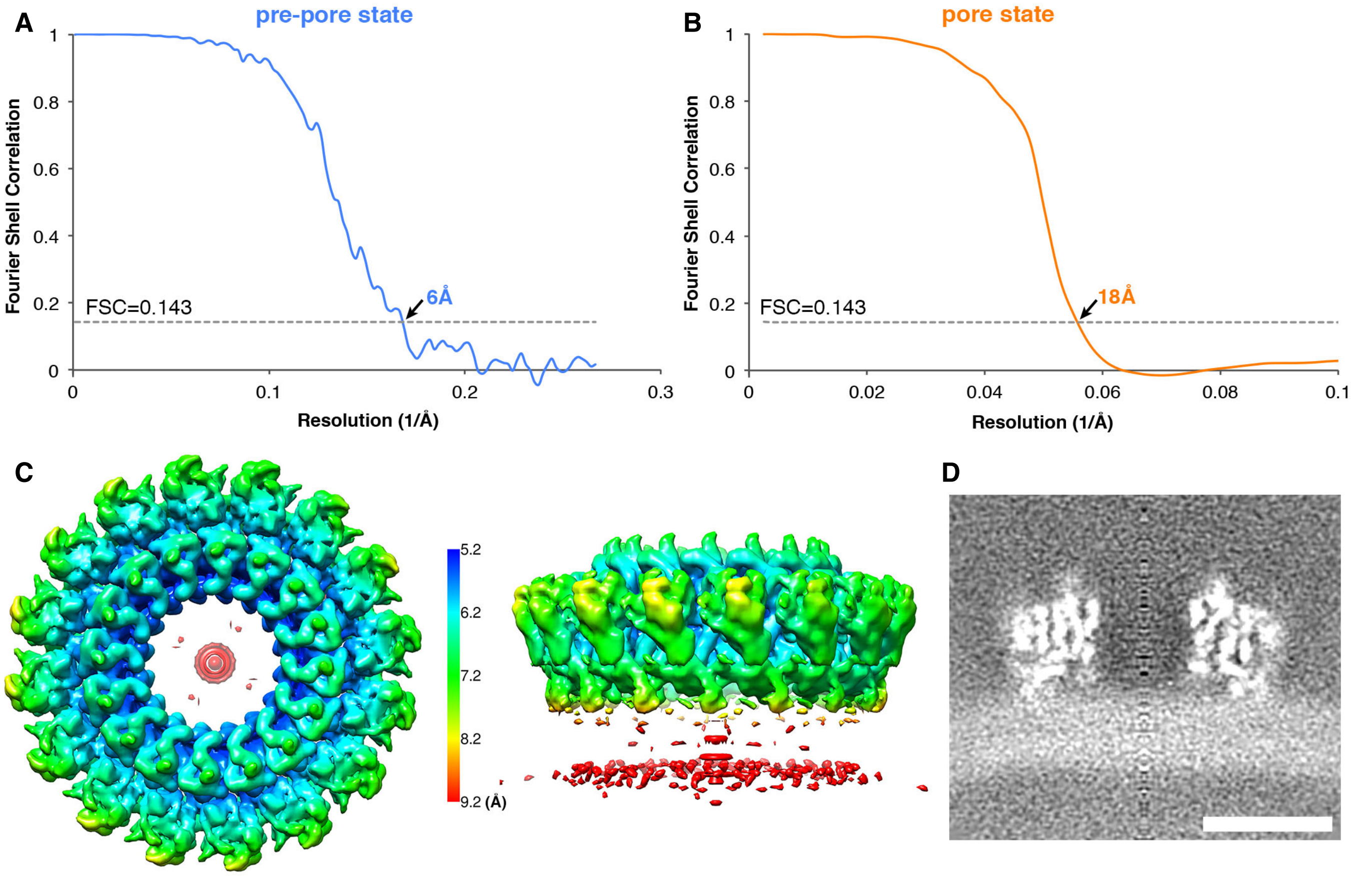

Figure EV1. Gold standard half map FSC of mPFN2 pre-pore and pore from subtomogram averaging.

A FSC of mPFN2 pre-pore on membrane.
B FSC of mPFN2 pore on membrane.
C Local resolution of mPFN2 pre-pore estimated in Relion.
D Central slice of mPFN2 pre-pore map in a sideview showing smearing of membrane density under mPFN2 pre-pore. Scale bar = 10 nm.

© 2022 The Authors

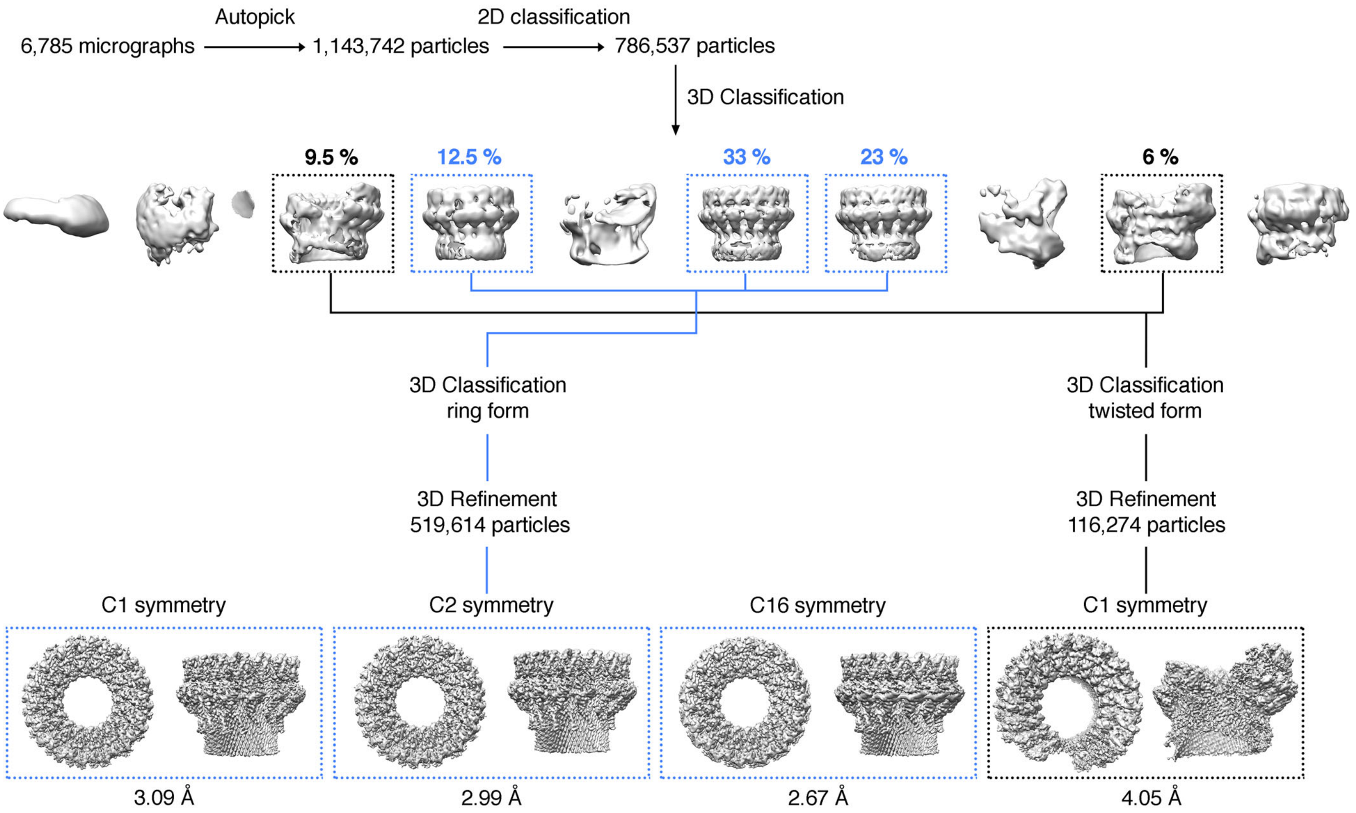

Figure EV2. Cryo-EM data processing workflow for mPFN2 pore structures in isolation.

The diagram illustrates the image processing and 3D reconstructions for mPFN2 pore in different conformations. Maps from the C2 symmetry in the flat ring conformation were used to generate the final map because the bottom of the β-barrel does not follow C16 symmetry.

© 2022 The Authors

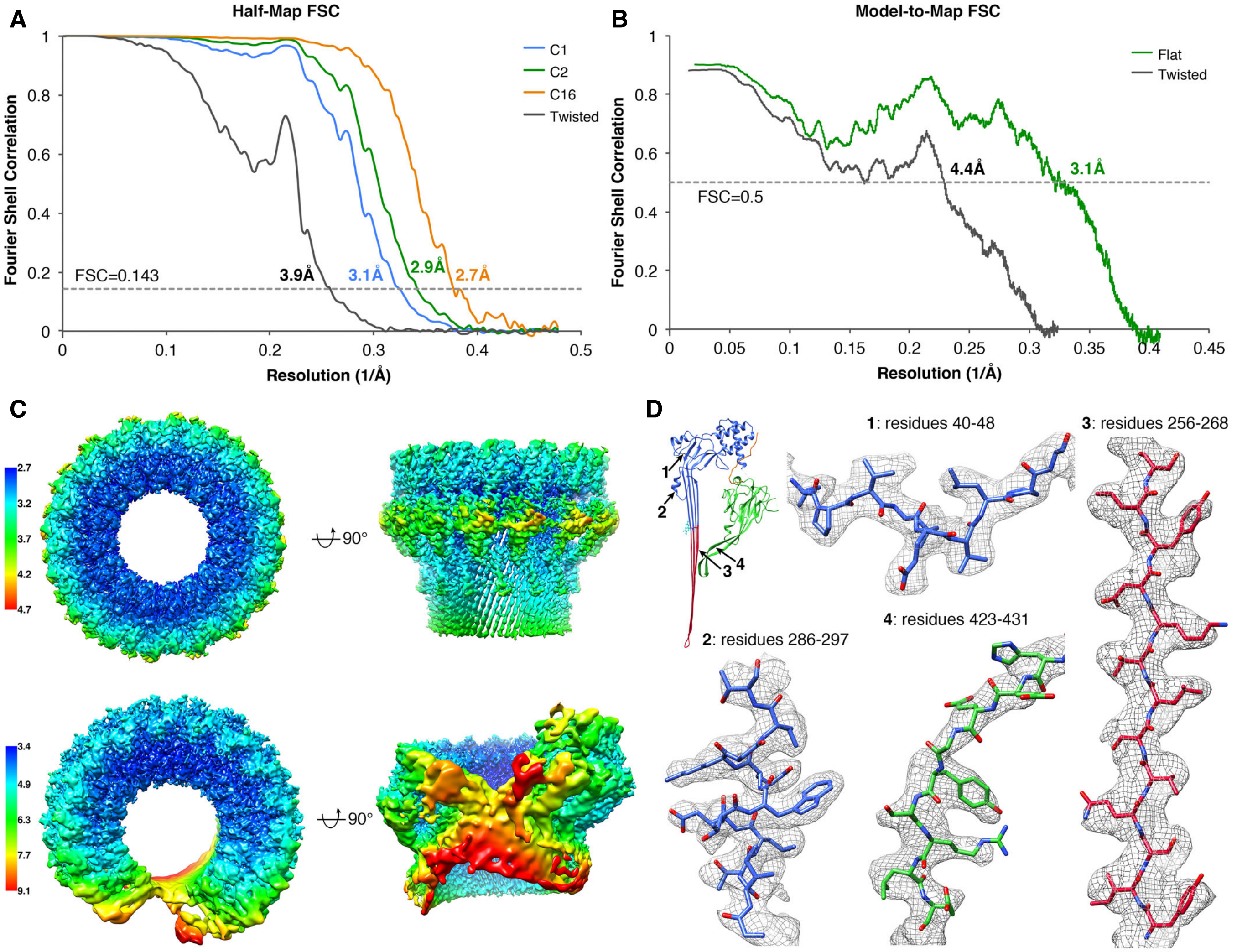

Figure EV3. Cryo-EM single particle analysis of mPFN2 pore structures in isolation.

A Gold-standard FSC curves of flat ring pore maps refined with C1, C2 and C16 symmetries and the twisted pore map with C1 symmetry. Soft masks were applied to two half-maps in relion postprocessing.

B Model-to-Map FSC curves showing the correlations between the refined atomic models and their corresponding cryo-EM maps.

C Local resolution of the mPFN2 pore in the ring (upper) and twisted (lower) conformations.

D Representative regions of cryo-EM densities (gray mesh) superposed with an atomic model of the mPFN2 pore in ring conformation and colored as in Fig 2E.

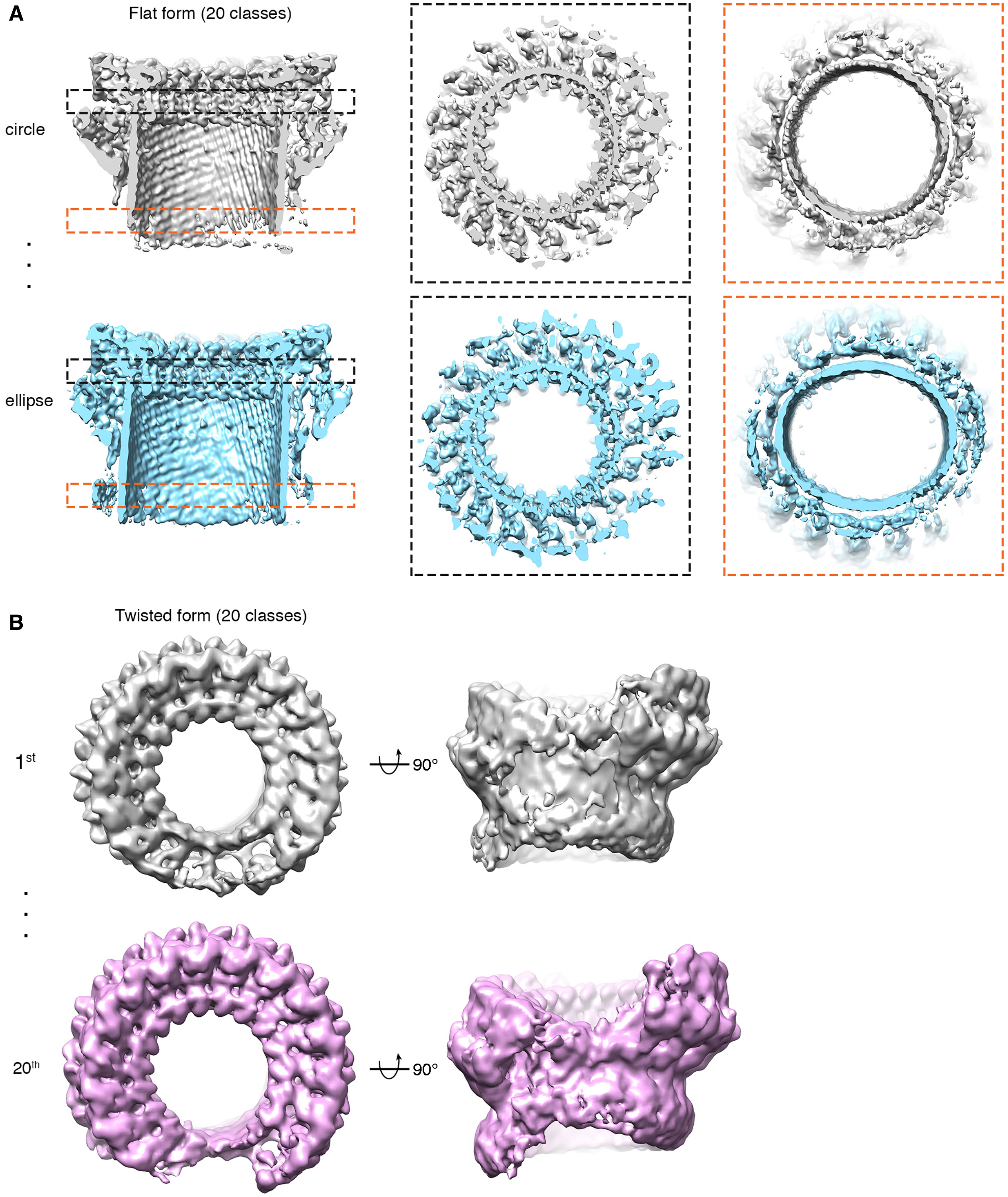

Figure EV4. 3D variation analysis of mPFN2 pore structures in isolation. Twenty classes were generated for both flat and twisted pores.

A Two representative 3D classes of mPFN2 ring conformations showing the β-barrel varies between circular and elliptical forms. Black dashed boxes indicate the consistency of circularity in the top of MACPF domain, while the orange dashed boxes show the variability in the bottom of the MACPF domain.

B The 1st and 20th 3D classes of mPFN2 twisted forms viewed from the top and the side, showing the variability of twist.

 © 2022 The Authors

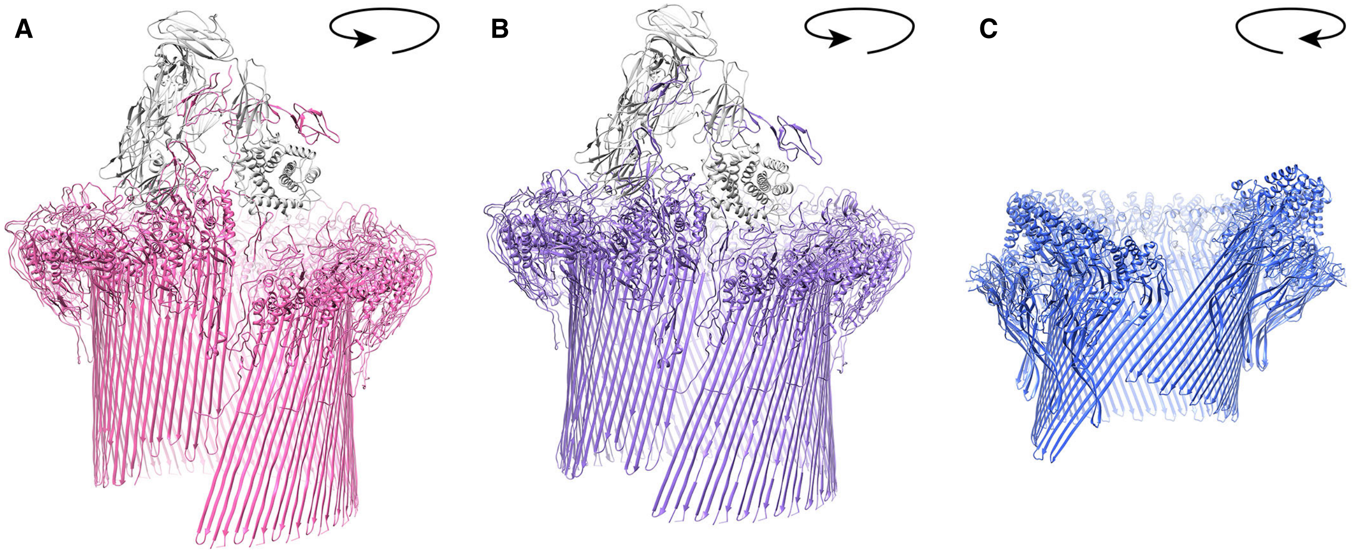

Figure EV5. Comparison of MAC and mPFN2 pores.

A, B Structures of MAC in two different conformations (PDB: 6H03 and 6H04).
C Structure of mPFN2 in the twisted pore conformation. The direction of twist is indicated by the arrows.
