# Supplementary material for: Cryo‐EM structures of perforin‐2 in isolation and assembled on a membrane suggest a mechanism for pore formation
Source: EMBO J. 2022 Oct 17;41(23):e111857. doi: 10.15252/embj.2022111857 (PMC9713709; doi:10.15252/embj.2022111857)
Supplement: Supplementary file 3 — Table EV1 [file EMBJ-41-e111857-s001.docx]

**Table EV1. Cryo-EM and cryo-ET data collection, refinement and validation statistics**

|  | #1  mPFN2 flat | #2  mPFN2 twisted | #3  mPFN2 pre-pore oligomer | #4  mPFN2 on liposome (pH7.5) | #5  mPFN2 on liposome (pH3.8) |
| --- | --- | --- | --- | --- | --- |
| **Data collection and processing** |  |  |  |  |  |
| Nominal Magnification | 130k | 130k | 120k | 64k | 53k |
| Voltage (kV) | 300 | 300 | 200 | 300 | 300 |
| Detector | Gatan K2 | Gatan K2 | Falcon 3 | Falcon 4 | Gatan K3 |
| Energy filter width | 20 eV | 20 eV | N.A. | 20 eV | 20 eV |
| Electron exposure (e–/Å^2^) | 41.7 | 41.7 | 40 | ~123 | ~123 |
| Defocus range (μm) | -1.0 ~ -2.4 | -1.0 ~ -2.4 | -1 ~ -3 | -2.5 ~ -5 | -3 ~ -6 |
| Pixel size (Å) | 1.047 | 1.047 | 1.218 | 1.875 | 1.63 |
| Symmetry imposed | C2 | C1 |  | C16 | C1 |
| Micrographs (tilt-series) |  |  |  |  |  |
| Initial particle images (no.) | 786,537 | 786,537 |  | 25,530 | 1369 |
| Final particle images (no.) | 519,614 | 116,274 |  | 3,695 | 992 |
| Map resolution (Å) | 3.0 | 4.0 |  | 6.0 | 18 |
| FSC threshold | 0.143 | 0.143 |  | 0.143 | 0.143 |
| Map resolution range (Å) | 2.7-4.7 | 3.4-9.1 |  | 5.2-9.2 |  |
|  |  |  |  |  |  |
| **Refinement** |  |  |  |  |  |
| Initial model (PDB code) | 6SB5 | 6SB5 |  |  |  |
| Model resolution (Å) |  |  |  |  |  |
| FSC threshold | 0.5 | 0.5 |  |  |  |
| Model resolution range (Å) | 3.1 | 6.2 |  |  |  |
| Map sharpening *B* factor (Å^2^) | -72 | -93 |  |  |  |
| Model composition |  |  |  |  |  |
| Non-hydrogen atoms | 67152 | 65592 |  |  |  |
| Protein residues | 8528 | 8528 |  |  |  |
| Ligands | 32 | 16 |  |  |  |
| *B* factors (Å^2^) |  |  |  |  |  |
| Protein | 32.87 | 104.46 |  |  |  |
| Ligand | 97.06 | 65.42 |  |  |  |
| R.m.s. deviations |  |  |  |  |  |
| Bond lengths (Å) | 0.007 | 0.009 |  |  |  |
| Bond angles (°) | 0.723 | 0.893 |  |  |  |
| Validation |  |  |  |  |  |
| MolProbity score | 1.81 | 2.0 |  |  |  |
| Clashscore | 10.18 | 15.72 |  |  |  |
| Poor rotamers (%) | 0.96 | 0.31 |  |  |  |
| Ramachandran plot |  |  |  |  |  |
| Favored (%) | 95.97 | 95.64 |  |  |  |
| Allowed (%) | 3.85 | 4.23 |  |  |  |
| Disallowed (%) | 0.18 | 0.13 |  |  |  |
| Accession codes | (EMD-15072)  (PDB 8A1D) | (EMD-15086)  (PDB 8A1S) |  | (EMD-15076) | (EMD-15087) |
