# Supplementary material for: Cryo‐EM structures of perforin‐2 in isolation and assembled on a membrane suggest a mechanism for pore formation
Source: EMBO J. 2022 Oct 17;41(23):e111857. doi: 10.15252/embj.2022111857 (PMC9713709; doi:10.15252/embj.2022111857)
Supplement: Supplementary file 9 [file EMBJ-41-e111857-s003.pdf]

THE EMBO JOURNAL

Cryo-EM structures of perforin-2 in isolation and assembled on a membrane suggest a mechanism for pore formation

Xiulian Yu, Tao Ni, George Munson, Peijun Zhang, and Robert Gilbert
DOI: 10.15252/embj.2022111857

Corresponding authors: Robert Gilbert (robert.gilbert@magd.ox.ac.uk) , Tao Ni (tao.ni@strubi.ox.ac.uk), Peijun Zhang (peijun.zhang@strubi.ox.ac.uk)

Review Timeline:

Submission Date:	13th Jun 22
Editorial Decision:	7th Jul 22
Revision Received:	29th Jul 22
Accepted:	9th Sep 22

Editor: Ieva Gailite

Transaction Report:

(Note: With the exception of the correction of typographical or spelling errors that could be a source of ambiguity, letters and reports are not edited. Depending on transfer agreements, referee reports obtained elsewhere may or may not be included in this compilation. Referee reports are anonymous unless the Referee chooses to sign their reports.)

Thank you for submitting your manuscript for consideration by The EMBO Journal. I sincerely apologise for the protracted review process due to delays in review submission. We have now received two referee reports on your manuscript, which are included below for your information.

As you will see from the comments, both reviewers appreciate the work, while also indicating some points for improvement of the study. Based on these positive assessments, I would like to invite you to address the concerns raised by the reviewers in a revised manuscript. In particular, reviewer #2 indicates that further structure-based mutagenesis experiments to support the proposed mechanism or peforin-2 pore formation would substantially strengthen the manuscript. I would be happy to discuss this or any other aspects of the revision via email or phone/videoconferencing. Please let me know which option would suit you better.

We generally allow three months as standard revision time. As a matter of policy, competing manuscripts published during this period will not negatively impact on our assessment of the conceptual advance presented by your study. However, please contact me as soon as possible upon publication of any related work to discuss the appropriate course of action. Should you foresee a problem in meeting this three-month deadline, please let us know in advance and we may be able to grant an extension.

When preparing your letter of response to the referees' comments, please bear in mind that this will form part of the Review Process File and will therefore be available online to the community. For more details on our Transparent Editorial Process, please visit our website: https://www.embopress.org/page/journal/14602075/authorguide#transparentprocess. Please also see the attached instructions for further guidelines on preparation of the revised manuscript.

Please feel free to contact me if you have any further questions regarding the revision. Thank you for the opportunity to consider your work for publication. I look forward to receiving the revised manuscript.

Referee #1:

This manuscript presents the exciting missing puzzle piece in how the immune pore perforin 2/MPEG1 forms pores in lipid membranes. Previous structures showed that the membrane-binding domain and the pore-forming machinery of the MACPF are diametrically opposed, presenting the field two competing models for how PFN2 forms pores. Based on new data presented here, the authors disprove the previous hypothesis that PFN2 engages membranes in trans and explain how PFN2 overcomes the steric obstacles of converting between these two conformations. The data is sound and supports the conclusions of the manuscript.

The manuscript presents cryotomography structure of the ectodomain of murine perforin2 on liposomes at neutral pH. They show this complex is a heterogeneous assembly comprised of a range of arc-like oligomers. Using subtomogram averaging, the authors calculate a 6A resolution structure with 16-fold symmetry, broadly agreeing with the soluble perforin2 oligomer previously published. Some important differences lie in the membrane-binding domain with subtle differences in orientation of the membrane binding b-hairpin and the C-terminal tail CTT. While it is clear from Figure 1 the differences in the CTT between their new structure and that found the soluble hPFN2 oligomer, it is hard to judge the comparison of the membrane-binding b-hairpin (green figure 1d). In this panel, the loop is only partially covered by density. The authors say that the membrane-associated conformation resembles 1 of the 2 conformations of this loop present in the artificial system of the double-stacked rings (previously published structure), but it is hard to judge the comparison from the image provided. Maybe the authors could include a supplementary figure overlay to show the reader how the density is better modelled by one conformation over the other? Or even report the local CC for these two fits in this region.

Next, they went on to solve the structures of the mPFN2 pore at a significantly higher resolution than their previous work, which allowed the authors to derive new biological insight. They find 2 main conformations: flat and twisted, which show substantial flexibility as the b-barrel extends towards the membrane. During the pore transition, many other domains become flexible (ie EGF-like domain) to accommodate the large conformational changes required. The authors talk about a "melting" of the EGF-like domain. Could the authors elaborate on what is meant by this? Could this not just be due to flexibility of a rigid body domain that is simply too small to see or that gets averaged out in the symmetrisation? Why do you think the whole structure collapses? EGFs are structured largely by coordination of calcium ions, do you expect a calcium depletion to happened when the pore forms? I didn't notice EDTA in the sample prep conditions.

Faced with technical challenges of forming the pores on liposomes, they came up with a creative solution that used preformed oligomers added to acidified liposomes. Using cryoTM, they showed mPFN2 forms arc pores and circular pores on the membrane surface. Here is the definitive evidence that PFN2 engaging the membrane in cis rather than trans, as was previously proposed.

The cryo tomograms show the pores are very heterogeneous. I was wondering if they can distinguish/identify any twisted pores in the membrane? Is it possible to get any information on what percentage of membrane-inserted pores are twisted vs flat and compare that with the percentage split from pores formed in detergent? Or is there too much heterogeneity in stoichiometry to determine this?

Overall, the authors present an interesting and thoughtful analysis of their structure and how it fits in the landscape of other published results. I really liked a critical analysis of how sample preparation can impact the structural arrangements found in oligomeric assemblies. Due to technical challenges, all structure (previously published and this study lack the TM domain). Could the authors comment on how the TM domain of PFN2 fits into their model of pore formation? Does it somehow regulate formation of the oligomer?

I agree the twisted barrel enables the large-scale conformational changes needed to unfurl the MACPF b-hairpins in the target membrane. Is it possible that PFN2 might also be a growing pore like PFN1 and MAC, rather than an all at once transition from prepore oligomer to pore? Perhaps a critical nucleation unit of MACPFs undergo this flip simultaneously and new monomers grow onto this arc, sequentially flipping to propagate the pore?

I enjoyed reading the authors discussion on the role of membrane PFN2 pore formation. Their work will clearly advance the broader field of pore forming proteins. They explain
how electrostatic charges of the protein surface could contribute to lipid clearance during pore formation and link their data nicely with what was known in the CDC field. Here they also discover density for a detergent molecule stabilizing the pore which could indicate a broader role of the membrane in stabilizing these transitions.

Technical comments:
Map/model fsc at 0.5 for the twisted conformation is 6.2A but their reported map resolution is 4A. I would have expected these to more closely match as they do in the flat case. Can the authors comment on this?

Referee #2:

Here the authors present cryo-EM structures of mouse perforin-2 (mPFN2) pre-pores (including both incomplete arc form with variable subunits and complete 16-subunit ring form) on liposomes (prepared from E. coli lipid extract), and two different conformations of pores (flat versus twisted) in isolation. These findings are largely consistent with the authors' previous structure work on PFN2 pre-pore and pore in isolation, with the new addition of the structure of pre-pore state on liposomes and also higher resolution of the pore structures. The authors attempted to resolve the pore structure in liposomes but encountered difficulties. Instead, the authors reported low resolution (~18A) subtomogram averaging analysis of mPFN2 pore configurations in liposomes, showing that majority are incomplete arc form, but not complete ring shape on artificial liposomes (composed of

POPC/SM).
The structures are high quality and beautifully presented. Based on these structures (and previous ones), the authors proposed a model to explain pre-pore to pore transition. However, the study suffers from a complete lack of functional validations. No structurally based mutagenesis experiments were carried out. Without any validation data, it is a stretch to propose such a model. The manuscript will be better served by tuning down these speculation parts, and focusing on analysis and comparison of structures resolved here versus structures reported previously (including comparison with MACPF and other CDCs). Additional discussion needs to be added to address other alternative explanations for their observed structures and limitations on their structures and what can be draw from these structures.
The title needs to be toned down and the abstract needs to completely revised to focus on the structure analysis, rather than any speculative models.
In Figure 1B, authors show a range of pre-pores including 10-mer, 12-mer, 13mer and 16-mer. There is only incomplete pore in liposomes in Fig. 5, but no complete pore. Is it possible that the 16-mer complete ring pre-pore is actually a dead-end, which cannot convert to transmembrane pore?
In the cryo-ET experiments, only 50 nm polycarbonate membrane for liposome extrusion was used, which could provide a membrane environment with high membrane curvatures, would this impact the formation of PFN2 prepore/pore?
-

Referee #1:

This manuscript presents the exciting missing puzzle piece in how the immune pore perforin 2/MPEG1 forms pores in lipid membranes. Previous structures showed that the membrane-binding domain and the pore-forming machinery of the MACPF are diametrically opposed, presenting the field two competing models for how PFN2 forms pores. Based on new data presented here, the authors disprove the previous hypothesis that PFN2 engages membranes in trans and explain how PFN2 overcomes the steric obstacles of converting between these two conformations. The data is sound and supports the conclusions of the manuscript.

We appreciate with thanks the reviewer's positive comments on the manuscript.

The manuscript presents cryotomography structure of the ectodomain of murine perforin2 on liposomes at neutral pH. They show this complex is a heterogeneous assembly comprised of a range of arc-like oligomers. Using subtomogram averaging, the authors calculate a 6A resolution structure with 16-fold symmetry, broadly agreeing with the soluble perforin2 oligomer previously published. Some important differences lie in the membrane-binding domain with subtle differences in orientation of the membrane binding b-hairpin and the C-terminal tail CTT. While it is clear from Figure 1 (now Figure 2 due to editorial request) the differences in the CTT between their new structure and that found the soluble hPFN2 oligomer, it is hard to judge the comparison of the membrane-binding b-hairpin (green Figure 2d (previous version, Figure 1d). In this panel, the loop is only partially covered by density. The authors say that the membrane-associated conformation resembles 1 of the 2 conformations of this loop present in the artificial system of the double-stacked rings (previously published structure), but it is hard to judge the comparison from the image provided. Maybe the authors could include a supplementary figure overlay to show the reader how the density is better modelled by one conformation over the other? Or even report the local CC for these two fits in this region.

Thank you. We have now included an Appendix Figure S1A, which shows the overlay of the mPFN2 model β-hairpin fit into the density map at different thresholds. Structures of mPFN2 in isolation and on membrane are compared with the density map overlayed, to highlight the difference of these two structures at CTT and β-hairpin regions (Appendix Figure S1B). We have also included a superposition of the two different conformations found for the hPFN2 CTT in the double-stacked rings, to show how one of the conformations observed is markedly better fit to the density observed than the other (Appendix Figure S1C).

Next, they went on to solve the structures of the mPFN2 pore at a significantly higher resolution than their previous work, which allowed the authors to derive new biological insight. They find 2 main conformations: flat and twisted, which show substantial flexibility as the b-barrel extends towards the membrane. During the pore transition, many other domains become flexible (ie EGF-like domain) to accommodate the large conformational changes required. The authors talk about a "melting" of the EGF-like domain. Could the authors elaborate on what is meant by this? Could this not just be due to flexibility of a rigid body domain that is simply too small to see or that gets averaged out in the symmetrisation? Why do you think the whole structure collapses? EGFs are structured largely by coordination of calcium ions, do you expect a calcium depletion to happened when the pore forms? I didn't notice EDTA in the sample prep conditions.

The unresolved EGF-like domain indicates a flexibility of this region, or disassembly of the domain. Further analysis of this region with symmetry expansion and focused classification from the cryo-EM

SPA dataset did not reveal interpretable density corresponding to the EGF-like domain. We apologize for the inaccurate usage of trivial phrasing (“melting”) and have instead replaced it as follows: “The apparent flexibility of these regions may facilitate the substantial conformational change of mPFN2 during pore formation.”

Faced with technical challenges of forming the pores on liposomes, they came up with a creative solution that used preformed oligomers added to acidified liposomes. Using cryoTM, they showed mPFN2 forms arc pores and circular pores on the membrane surface. Here is the definitive evidence that PFN2 engaging the membrane in cis rather than trans, as was previously proposed.

We appreciate the reviewer’s positive comments, though we note that the sample preparation did not consist of fully-formed pre-pore assemblies. We believe it will have included a range of association states of which our analysis shown in Figure EV1a in the previous submission and now in Figure 1 (by editorial request) suggests the most populous may have been trimers. In any case we agree that membrane binding by mPFN2 from solution to create pores *in situ* does show in this case pore formation in *cis*.

The cryo tomograms show the pores are very heterogeneous. I was wondering if they can distinguish/identify any twisted pores in the membrane? Is it possible to get any information on what percentage of membrane-inserted pores are twisted vs flat and compare that with the percentage split from pores formed in detergent? Or is there too much heterogeneity in stoichiometry to determine this?

Due to problems with sample aggregation, we can only identify a limited number of pores on the liposomes. The heterogeneity in stoichiometry can be observed in the raw tomograms (Figure 6) (previous version, Figure 5) and the very limited number of subtomograms does not allow confident classification of the heterogenous pores on the liposome. We therefore cannot reliably distinguish/identify twisted pores in the membrane, though we have attempted to do this as part of our analysis pipeline.

Overall, the authors present an interesting and thoughtful analysis of their structure and how it fits in the landscape of other published results. I really liked a critical analysis of how sample preparation can impact the structural arrangements found in oligomeric assemblies. Due to technical challenges, all structure (previously published and this study) lack the TM domain. Could the authors comment on how the TM domain of PFN2 fits into their model of pore formation? Does it somehow regulate formation of the oligomer?

The PFN2 ectodomain is believed to be cleaved off from the TM domain during bacterial restriction, therefore we think it does not contribute to PFN2 pre-pore or pore formation directly for bactericidal activity. However, ubiquitination of the PFN2 cytosolic tail has been shown to be responsible for the recruitment of PFN2 from endosomes to bacterial compartments to confer bactericidal activity (McCormack *et al* 2015 Elife), suggesting the necessity of full-length protein (including the TM domain) for early-stage trafficking.

Furthermore, we cannot exclude the possibility that uncleaved membrane-anchored PFN2 is capable in pore formation. It is possible that the full-length PFN2 (rather than the PFN2 ectodomain) can already oligomerize on the host membrane where they are anchored by the TM (the flexible linker between the CTT domain and TM domain would allow the anchoring of the full-length PFN2 onto the host membrane in its assembled pre-pore form). Further investigation (such as structural analysis *in situ* during bacterial infection) is necessary to address this question. It is worth noting here that the report that IL33 is released by PFN2 from dendritic cells (Hung et al., 2020) could be another application of pore formation by full-length, membrane-anchored protein. We have inserted a few lines into the Discussion to note these points.

I agree the twisted barrel enables the large-scale conformational changes needed to unfurl the MACPF b-hairpins in the target membrane. Is it possible that PFN2 might also be a growing pore like PFN1

and MAC, rather than an all at once transition from prepore oligomer to pore? Perhaps a critical nucleation unit of MACPFs undergo this flip simultaneously and new monomers grow onto this arc, sequentially flipping to propagate the pore?

We did not observe evidence that PFN2 is a growing pore. Because the orientation of the MACPF domain in the pre-pore and pore forms are opposite in PFN2 (and the monomeric form seems to have a conformation like that of the pre-pore incorporated subunits; Figure 1 (previously Figure EV1), then if a nucleating PFN2 pore flips and inserts into the membrane first, the resulting structure (in the pore conformation) is unlikely to provide a supporting interface matching new monomers and enabling oligomeric growth. In contrast, MACPF domains in PFN1 and the MAC enable the possibility of a growing pore: MACPF in PFN1 pre-pore and pore are in the same orientation on the membrane; and C9 does not have a membrane binding domain to restrict its orientation before addition to the existing pore on the membrane.

In a related manuscript from Simon Scheuring's group (Jiao et al., 2022) we have described observation of a real-time transition of PFN2 from pre-pore to pore by atomic force microscopy, which showed a clockwise propagation of the associated conformational change. This paper has been resubmitted for the second time following reviewers' comments suggesting eventual successful publication at the handling journal – we now reference it at the end of our results in arguing for he model of pore formation shown in Figure 7 (previous version, Figure 6). While the AFM data do not argue directly against a growing pore model for PFN2, they suggest that transition from pre-pore to pore initiates from one or a few critical point(s), and facilitates transition of its/their neighbour subunit in a clockwise manner. We believe for reasons of the opposite orientation of the MACPF domain and its target membrane in the pre-pore state, and on the basis of the AFM data, that a growing pore mechanism is unlikely in PFN2.

I enjoyed reading the authors discussion on the role of membrane PFN2 pore formation. Their work will clearly advance the broader field of pore forming proteins. They explain how electrostatic charges of the protein surface could contribute to lipid clearance during pore formation and link their data nicely with what was known in the CDC field. Here they also discover density for a detergent molecule stabilizing the pore which could indicate a broader role of the membrane in stabilizing these transitions.

Again, we are grateful to the referee for their positive and thoughtful assessment of our manuscript.

Technical comments:
Map/model fsc at 0.5 for the twisted conformation is 6.2A but their reported map resolution is 4A. I would have expected these to more closely match as they do in the flat case. Can the authors comment on this?

We apologise for the error here: the map-to-model (FSC=0.5) has a resolution of 4.4 Å, as shown in the Figure EV2B (previously EV3B). We have now corrected the cryoEM data collection and analysis in Table S1.

Referee #2:

Here the authors present cryo-EM structures of mouse perforin-2 (mPFN2) pre-pores (including both incomplete arc form with variable subunits and complete 16-subunit ring form) on liposomes (prepared from E. coli lipid extract), and two different conformations of pores (flat versus twisted) in isolation. These findings are largely consistent with the authors' previous structure work on PFN2 pre-pore and pore in isolation, with the new addition of the structure of pre-pore state on liposomes and also higher resolution of the pore structures. The authors attempted to resolve the pore structure in liposomes but encountered difficulties. Instead, the authors reported low resolution (~18A)

subtomogram averaging analysis of mPFN2 pore configurations in liposomes, showing that majority are incomplete arc form, but not complete ring shape on artificial liposomes (composed of POPC/SM).

We are grateful to referee #2 also for their positive assessment of our work.

The structures are high quality and beautifully presented. Based on these structures (and previous ones), the authors proposed a model to explain pre-pore to pore transition. However, the study suffers from a complete lack of functional validations. No structurally based mutagenesis experiments were carried out. Without any validation data, it is a stretch to propose such a model.

We agree with the referee that such a remarkable model for pore formation must benefit from functional validation, such as structurally-based mutagenesis. When we were first proposing our model for the pore formation mechanism of PFN2 in Ni et al., 2020 we therefore did provide some supporting mutagenic data, using disulphide locking of the MACPF and P2 domain orientations to infer that, without MACPF domain rotation, pore formation could not occur (Ni et al., 2020 figure S7). In addition to locking experiments, and mutagenesis revealing that the P2 domain is indeed responsible for membrane binding (Ni et al., 2020 figure 2), we can now point to a companion paper by Simon Scheuring and colleagues with ourselves which demonstrates using high-speed AFM the dynamics of the pre-pore to pore transition, including the clockwise propagation of the conformational change involved which we have not emphasised in our paper but which is implied by our findings and in Figure 7 (previously Figure 6). We have included in the resubmitted manuscript a direct reference to the previously-published mutagenic data and also to the paper by Scheuring et al., at the end of the Results section where we are presenting our model for the pore-forming transition with Figure 7 (previously Figure 6).

The manuscript will be better served by tuning down these speculation parts, and focusing on analysis and comparison of structures resolved here versus structures reported previously (including comparison with MACPF and other CDCs).

We have now provided a supplementary figure (Appendix Figure S2) to compare the structures of the pore-forming regions of MACPF domain-containing proteins including CDCs, and revised our paper to emphasise the evidential basis for the model we present.

Additional discussion needs to be added to address other alternative explanations for their observed structures and limitations on their structures and what can be draw from these structures.

In the revised manuscript we now ensure we consider alternative explanations for the observations we make, including the possible role of different mechanisms for pore formation in different contexts and of full-length (membrane-anchored) PFN2 in pore formation (pages 8 and 9).

The title needs to be toned down and the abstract needs to completely revised to focus on the structure analysis, rather than any speculative models.

The title has been changed and the abstract rewritten to emphasise the data actually observed and then to suggest the interpretation we favour.

In Figure 1B, authors show a range of pre-pores including 10-mer, 12-mer, 13mer and 16-mer. There is only incomplete pore in liposomes in Fig. 5, but no complete pore. Is it possible that the 16-mer complete ring pre-pore is actually a dead-end, which cannot convert to transmembrane pore?

This now refers to Figures 2B and 6 in the resubmitted version. We don't think that 16-mer complete ring pre-pore is a dead-end product because (1) the 16-mer pre-pore in isolation can convert to 16-mer pore, as described in the sample preparation for the isolated pore: "pore-forming assemblies were prepared from pre-pore oligomers pre-assembled at pH 5.5, in which condition the bulk of the oligomers are complete full rings (Ni et al., 2020), and subsequently by lowering the pH to 3.6-4.0 in the presence of 0.056% Cymal-6 (Ni et al., 2020)"; (2) a related manuscript from Simon Scheuring's

group shows real-time transition of ring pre-pores to pores on membranes by atomic force microscopy. This paper is a significant addition to the observations by AFM of pre-pore to pore transition by full rings made in our previous publication, Ni et al., 2020, and is mentioned at the end of the Results section.

In the cryo-ET experiments, only 50 nm polycarbonate membrane for liposome extrusion was used, which could provide a membrane environment with high membrane curvatures, would this impact the formation of PFN2 prepore/pore?

We observed variable sized liposomes with PFN2 pre-pore bound in the prep, as shown in Fig 2A (previously, Figure 1A). We also tried 100 nm polycarbonate membranes for liposome extrusion previously and found PFN2 pre-pore and pore assembly on the membrane (Figure S6 in Ni et al., 2020). It is possible that membrane curvature may affect formation of PFN2 oligomers. However, a systematic comparison would be necessary to understand the impact of membrane curvature to PFN2 oligomer formation, which is beyond the scope of the current study. However, we have added a brief reference to this interesting question (pages 8-9), which has been a matter of comment in the literature for MACPF/CDC proteins for many years.

With best wishes

Robert Gilbert (on behalf of all authors)

1st Revision - Editorial Decision 9th Sep 2022

Thank you for submitting a revised version of your manuscript. Your study has now been seen by both original referees, who find that their concerns have been addressed and recommend publication of the manuscript. I am therefore pleased to inform you that your manuscript has been accepted for publication in The EMBO Journal.

There remain only a couple of issues that have to be addressed before I can transfer your manuscript to our publishers at Wiley for typesetting:

1. We are missing the ORCID iD for the co-corresponding author Peijun Zhang. In order to link the ORCID iD to the account in our manuscript tracking system, the author in question has to do the following:
- Click the 'Modify Profile' link at the bottom of your homepage in our system.
- On the next page you will see a box halfway down the page titled ORCID*. Below this box is red text reading 'To Register/Link to ORCID, click here'. Please follow that link: you will be taken to ORCID where you can log in to your account (or create an account if you don't have one)
- You will then be asked to authorise Wiley to access your ORCID information. Once you have approved the linking, you will be brought back to our manuscript system.

Unfortunately we cannot do this linking on the author's behalf for security reasons.

2. Papers published in The EMBO Journal are accompanied online by a 'Synopsis' to enhance discoverability of the manuscript. It consists of A) a short (1-2 sentences) summary of the findings and their significance, B) 3-4 bullet points highlighting key results and C) a synopsis image that is 550x300-600 pixels large (width x height, jpeg or png format). You can either show a model or key data in the synopsis image. Please note that the size is rather small and that text needs to be readable at the final size. Please send me your proposal for the synopsis text and the accompanying image.

Please note that it is EMBO Journal policy for the transcript of the editorial process (containing referee reports and your response letter) to be published as an online supplement to each paper. If you do NOT want this, you will need to inform the Editorial Office via email immediately. More information is available here:
https://www.embopress.org/page/journal/14602075/authorguide#transparentprocess

Your manuscript will be processed for publication in the journal by EMBO Press. Manuscripts in the PDF and electronic editions of The EMBO Journal will be copy edited, and you will be provided with page proofs prior to publication. Please note that supplementary information is not included in the proofs.

You will be contacted by Wiley Author Services to complete licensing and payment information. The required 'Page Charges Authorization Form' is available here: https://www.embopress.org/pb-assets/embo-site/tej_apc.pdf - please download and complete the form and return to embopressproduction@wiley.com

Should you be planning a Press Release on your article, please get in contact with embojournal@wiley.com as early as possible, in order to coordinate publication and release dates.

Finally, under the agreement of the UK research institutions with our publisher Wiley, you could be eligible for free publication of your article in the open access format (https://authorservices.wiley.com/author-resources/Journal-Authors/open-access/affiliation-policies-payments/jisc-agreement.html). Please contact either the administration at your institution or our publishers at Wiley (embojournal@wiley.com) for further questions.

Thank you again for this contribution to The EMBO Journal and congratulations on a successful publication!

Referee #1:

all comments/issues have been addressed.

Referee #2:

The authors' revision is comprehensive and satisfactory. I have no further questions - beautiful work.

EMBO Press Author Checklist

Corresponding Author Name: Robert Gilbert	
Journal Submitted to: EMBO J	
Manuscript Number: EMBOJ-2022-111857	

USEFUL LINKS FOR COMPLETING THIS FORM
The EMBO Journal - Author Guidelines
EMBO Reports - Author Guidelines
Molecular Systems Biology - Author Guidelines
EMBO Molecular Medicine - Author Guidelines

Reporting Checklist for Life Science Articles (updated January 2022)
This checklist is adapted from Materials Design Analysis Reporting (MDAR) Checklist for Authors. MDAR establishes a minimum set of requirements in transparent reporting in the life sciences (see Statement of Task: 10.31222/osf.io/9sm4x). Please follow the journal's guidelines in preparing your manuscript.
Please note that a copy of this checklist will be published alongside your article.

Abridged guidelines for figures
1. Data
The data shown in figures should satisfy the following conditions:
- the data were obtained and processed according to the field's best practice and are presented to reflect the results of the experiments in an accurate and unbiased manner.
- ideally, figure panels should include only measurements that are directly comparable to each other and obtained with the same assay.
- plots include clearly labeled error bars for independent experiments and sample sizes. Unless justified, error bars should not be shown for technical replicates.
- if n<5, the individual data points from each experiment should be plotted. Any statistical test employed should be justified.
- Source Data should be included to report the data underlying figures according to the guidelines set out in the authorship guidelines on Data Presentation.

2. Captions
Each figure caption should contain the following information, for each panel where they are relevant:
- a specification of the experimental system investigated (eg cell line, species name).
- the assay(s) and method(s) used to carry out the reported observations and measurements.
- an explicit mention of the biological and chemical entity(ies) that are being measured.
- an explicit mention of the biological and chemical entity(ies) that are altered/varied/perturbed in a controlled manner.
- the exact sample size (n) for each experimental group/condition, given as a number, not a range;
- a description of the sample collection allowing the reader to understand whether the samples represent technical or biological replicates (including how many animals, litters, cultures, etc.).
- a statement of how many times the experiment shown was independently replicated in the laboratory.
- definitions of statistical methods and measures:
  - common tests, such as t-test (please specify whether paired vs. unpaired), simple χ2 tests, Wilcoxon and Mann-Whitney tests, can be unambiguously identified by name only, but more complex techniques should be described in the methods section;
  - are tests one-sided or two-sided?
  - are there adjustments for multiple comparisons?
  - exact statistical test results, e.g., P values = x but not P values < x;
  - definition of 'center values' as median or average;
  - definition of error bars as s.d. or s.e.m.

Please complete ALL of the questions below.
Select "Not Applicable" only when the requested information is not relevant for your study.

Materials

Newly Created Materials	Information included in the manuscript?	In which section is the information available? (Reagents and Tools Table, Materials and Methods, Figures, Data Availability Section)
New materials and reagents need to be available; do any restrictions apply?	Not Applicable	

Antibodies	Information included in the manuscript?	In which section is the information available? (Reagents and Tools Table, Materials and Methods, Figures, Data Availability Section)
For **antibodies** provide the following information:<br>- Commercial antibodies: RRID (if possible) or supplier name, catalogue number and or/clone number<br>- Non-commercial: RRID or citation	Not Applicable	

DNA and RNA sequences	Information included in the manuscript?	In which section is the information available? (Reagents and Tools Table, Materials and Methods, Figures, Data Availability Section)
**Short novel DNA or RNA including primers, probes**: provide the sequences.	Not Applicable	

Cell materials	Information included in the manuscript?	In which section is the information available? (Reagents and Tools Table, Materials and Methods, Figures, Data Availability Section)
**Cell lines:** Provide species information, strain. Provide accession number in repository **OR** supplier name, catalog number, clone number, and/**OR** RRID.	Yes	Materials and Methods
**Primary cultures:** Provide species, strain, sex of origin, genetic modification status.	Not Applicable	
Report if the cell lines were recently **authenticated** (e.g., by STR profiling) and tested for mycoplasma contamination.	Not Applicable	

Experimental animals	Information included in the manuscript?	In which section is the information available? (Reagents and Tools Table, Materials and Methods, Figures, Data Availability Section)
**Laboratory animals or Model organisms:** Provide species, strain, sex, age, genetic modification status. Provide accession number in repository **OR** supplier name, catalog number, clone number, **OR** RRID.	Not Applicable	
**Animal observed in or captured from the field:** Provide species, sex, and age where possible.	Not Applicable	
Please detail **housing and husbandry conditions**.	Not Applicable	

Plants and microbes	Information included in the manuscript?	In which section is the information available? (Reagents and Tools Table, Materials and Methods, Figures, Data Availability Section)
**Plants:** provide species and strain, ecotype and cultivar where relevant, unique accession number if available, and source (including location for collected wild specimens).	Not Applicable	
**Microbes:** provide species and strain, unique accession number if available, and source.	Not Applicable	

Human research participants	Information included in the manuscript?	In which section is the information available? (Reagents and Tools Table, Materials and Methods, Figures, Data Availability Section)
If collected and within the bounds of privacy constraints report on age, sex and gender or ethnicity for all study participants.	Not Applicable	

Core facilities	Information included in the manuscript?	In which section is the information available? (Reagents and Tools Table, Materials and Methods, Figures, Data Availability Section)
If your work benefited from core facilities, was their service mentioned in the acknowledgments section?	Yes	Materials and Methods, Acknowledgements

Design

Study protocol	Information included in the manuscript?	In which section is the information available? (Reagents and Tools Table, Materials and Methods, Figures, Data Availability Section)
If study protocol has been **pre-registered, provide DOI in the manuscript**. For clinical trials, provide the trial registration number **OR** cite DOI.	Not Applicable	
Report the **clinical trial registration number** (at ClinicalTrials.gov or equivalent), where applicable.	Not Applicable	

Laboratory protocol	Information included in the manuscript?	In which section is the information available? (Reagents and Tools Table, Materials and Methods, Figures, Data Availability Section)
Provide DOI OR other citation details if **external detailed step-by-step protocols** are available.	Not Applicable	

Experimental study design and statistics	Information included in the manuscript?	In which section is the information available? (Reagents and Tools Table, Materials and Methods, Figures, Data Availability Section)
Include a statement about **sample size** estimate even if no statistical methods were used.	Not Applicable	
Were any steps taken to minimize the effects of subjective bias when allocating animals/samples to treatment (e.g. **randomization procedure**)? If yes, have they been described?	Not Applicable	
Include a statement about **blinding** even if no blinding was done.	Not Applicable	
Describe **inclusion/exclusion criteria** if samples or animals were excluded from the analysis. Were the criteria pre-established?<br><br>If sample or data points were omitted from analysis, report if this was due to attrition or intentional exclusion and provide justification.	Not Applicable	
For every figure, are **statistical tests** justified as appropriate? Do the data meet the assumptions of the tests (e.g., normal distribution)? Describe any methods used to assess it. Is there an estimate of variation within each group of data? Is the variance similar between the groups that are being statistically compared?	Yes	Results, Materials and Methods

Sample definition and in-laboratory replication	Information included in the manuscript?	In which section is the information available? (Reagents and Tools Table, Materials and Methods, Figures, Data Availability Section)
In the figure legends: state number of times the experiment was **replicated** in laboratory.	Not Applicable	
In the figure legends: define whether data describe **technical or biological replicates**.	Not Applicable	

Ethics

Ethics	Information included in the manuscript?	In which section is the information available? (Reagents and Tools Table, Materials and Methods, Figures, Data Availability Section)
Studies involving **human participants**: State details of **authority granting ethics approval** (IRB or equivalent committee(s), provide reference number for approval.	Not Applicable	
Studies involving **human participants**: Include a statement confirming that **informed consent** was obtained from all subjects and that the experiments conformed to the principles set out in the WMA Declaration of Helsinki and the Department of Health and Human Services Belmont Report.	Not Applicable	
Studies involving **human participants:** For publication of **patient photos**, include a statement confirming that consent to publish was obtained.	Not Applicable	
Studies involving experimental **animals**: State details of **authority granting ethics approva**l (IRB or equivalent committee(s), provide reference number for approval. Include a statement of compliance with ethical regulations.	Not Applicable	
Studies involving **specimen and field samples:** State if relevant **permits** obtained, provide details of authority approving study; if none were required, explain why.	Not Applicable	

Dual Use Research of Concern (DURC)	Information included in the manuscript?	In which section is the information available? (Reagents and Tools Table, Materials and Methods, Figures, Data Availability Section)
Could your study fall under dual use research restrictions? Please check biosecurity documents and list of **select agents and toxins** (CDC): https://www.selectagents.gov/sat/list.htm	Not Applicable	
If you used a select agent, is the security level of the lab appropriate and reported in the manuscript?	Not Applicable	
If a study is subject to dual use research of concern regulations, is the name of the **authority granting approval and reference number** for the regulatory approval provided in the manuscript?	Not Applicable	

Reporting

The MDAR framework recommends adoption of discipline-specific guidelines, established and endorsed through community initiatives. Journals have their own policy about requiring specific guidelines and recommendations to complement MDAR.

Adherence to community standards	Information included in the manuscript?	In which section is the information available? (Reagents and Tools Table, Materials and Methods, Figures, Data Availability Section)
State if relevant guidelines or checklists (e.g., **ICMJE, MIBBI, ARRIVE, PRISMA**) have been followed or provided.	Not Applicable	
For **tumor marker prognostic studies,** we recommend that you follow the **REMARK** reporting guidelines (see link list at top right). See author guidelines, under 'Reporting Guidelines'. Please confirm you have followed these guidelines.	Not Applicable	
For **phase II and III randomized controlled trials**, please refer to the **CONSORT** flow diagram (see link list at top right) and submit the CONSORT checklist (see link list at top right) with your submission. See author guidelines, under 'Reporting Guidelines'. Please confirm you have submitted this list.	Not Applicable	

Data Availability

Data availability	Information included in the manuscript?	In which section is the information available? (Reagents and Tools Table, Materials and Methods, Figures, Data Availability Section)
Have **primary datasets** been deposited according to the journal's guidelines (see 'Data Deposition' section) and the respective accession numbers provided in the Data Availability Section?	Yes	Data Availability Section
Were **human clinical and genomic datasets** deposited in a public access-controlled repository in accordance to ethical obligations to the patients and to the applicable consent agreement?	Not Applicable	
Are **computational models** that are central and integral to a study available without restrictions in a machine-readable form? Were the relevant accession numbers or links provided?	Yes	Data Availability Section
If publicly available data were reused, provide the respective **data citations in the reference list**.	Not Applicable	
